# Effects of 12 weeks of Tai Chi on neuromuscular responses and postural control in elderly patients with sarcopenia: a randomized controlled trial

**DOI:** 10.3389/fneur.2023.1167957

**Published:** 2023-04-28

**Authors:** Dunbing Huang, Xiaohua Ke, Cai Jiang, Wei Song, Jing Feng, Huiting Zhou, Rui Zhang, Anren Zhang, Fujun Lan

**Affiliations:** ^1^Department of Rehabilitation Medicine, Shanghai Fourth People’s Hospital, School of Medicine, Tongji University, Shanghai, China; ^2^Shengli Clinical Medical College, Fujian Medical University, Fuzhou, China; ^3^The Second Rehabilitation Department, Fujian Provincial Hospital, Fuzhou, China; ^4^Fujian Institute of Clinical Geriatric, Fujian Provincial Hospital, Fuzhou, China; ^5^Department of Rehabilitation Medicine, Zhejiang Hospital, Hangzhou, China; ^6^Department of Neurosurgery, Zhejiang Hospital, Hangzhou, China

**Keywords:** Tai Chi, neuromuscular responses, postural control, elderly patients, sarcopenia

## Abstract

**Objective:**

To explore the effect of 12 weeks of Tai Chi on neuromuscular responses and postural control in elderly patients with sarcopenia.

**Methods:**

One hundred and twenty-four elderly patients with sarcopenia from ZheJiang Hospital and surrounding communities were selected, however, 64 were later disqualified. Sixty elderly patients with sarcopenia were randomly assigned to the Tai Chi group (*n* = 30) and the control group (*n* = 30). Both groups received 45-min health education sessions once every 2 weeks for 12 weeks, and the Tai Chi group engaged in 40-min simplified eight-style Tai Chi exercise sessions 3 times per week for 12 weeks. Two assessors who had received professional training and were unaware of the intervention allocation assessed the subjects within 3 days prior to the intervention and within 3 days after completion of the intervention. They chose the unstable platform provided by the dynamic stability test module in ProKin 254 to evaluate the patient’s postural control ability. Meanwhile, surface EMG was utilized to assess the neuromuscular response during this period.

**Results:**

After 12 weeks of intervention, the Tai Chi group showed a significant decrease in neuromuscular response times of the rectus femoris, semitendinosus, anterior tibialis, and gastrocnemius and overall stability index (OSI) compared to before the intervention (*p* < 0.05), while there was no significant difference in the control group for these indicators before and after intervention (*p >* 0.05). In addition, these indicators in the Tai Chi group were significantly lower than those in the control group (*p* < 0.05). The changes in neuromuscular response times of the rectus femoris, semitendinosus, anterior tibialis, and gastrocnemius were positively correlated with the changes in OSI (*p* < 0.05) in the Tai Chi group, but there were no significant correlations between changes in neuromuscular response times of the aforementioned muscles and changes in OSI in the control group (*p* < 0.05).

**Conclusion:**

Twelve-weeks of Tai Chi exercise can improve the neuromuscular response of the lower extremities in elderly patients with sarcopenia, shorten their neuromuscular response time when balance is endangered, enhance their dynamic posture control ability, and ultimately reduce the risk of falls.

## Introduction

Sarcopenia is a clinical syndrome characterized by a progressive and widespread loss of skeletal muscle mass and/or muscle strength related to the aging process (1). As reported by the Asian Working Group for Sarcopenia, nearly 1/3 of people aged 65 years and older suffer from sarcopenia, and the prevalence is as high as 50–60% in people aged 80 years and older (2). It is predicted that over the course of the next 40 years, up to 200 million people in mainland China will suffer from this illness (3). Sarcopenia causes a decrease in muscle mass and muscle strength, which reduces mobility in elderly individuals and is one of the main physiological factors contributing to falls in elderly individuals (4). Regardless of whether falls cause physical or psychological damage to older adults, they can limit the daily mobility and functional activities of older adults and exacerbate the loss of skeletal muscle mass and muscle strength, thus further limiting their ability to perform daily activities and ultimately leading to a reduced quality of life and even death (5, 6). It has been reported that the annual probability of falls in the general elderly population over 60 years of age is 20.7% (7), while the incidence of falls in the elderly population with sarcopenia is two to three times higher than that of the general elderly population (8). In addition, studies have shown that the mortality rate of elderly people with sarcopenia due to falls is one to five times higher than that of normal elderly people (9). In conclusion, elderly patients with sarcopenia face serious fall risks and accidental injuries from falls.

Regular exercise has been indicated to improve physical and mental health (such as reducing anxiety and depression and boosting self-confidence); reduce the risk of developing diseases (such as heart disease, diabetes, and stroke) and mortality; increase social participation, integration, and adaptation; and prevent falls and fall-related injuries. In particular, Battaglia et al. demonstrated that the adaptation of the body, such as increased support surface and equal redistribution of body weight on both feet, resulting from a 5-week dynamic balance training protocol, may be enough to improve static balance in elderly women (10). Won et al. found that regular exercise can enhance the subjective well-being, life satisfaction, leisure satisfaction, and exercise satisfaction of older adults and improve their quality of life (11). Similarly, Korniloff et al. found that exercise can reduce the risk of depression in older people, and the frequency of exercise is negatively correlated with the incidence of depression (12). Exercise is the most economical and effective way to prevent and improve sarcopenia in the elderly in the long term. However, resistance exercises are often characterized by high intensity and fast pace, making it difficult for elderly individuals with sarcopenia to complete them. Additionally, high-intensity resistance exercises can easily induce cardiovascular and cerebrovascular diseases. Although aerobic exercise can improve the cardiorespiratory function and activity level of elderly individuals, there is still controversy about the impact of aerobic exercise on muscle mass and muscle strength in elderly individuals (13). Therefore, it is imperative to find appropriate clinical interventions to reduce the risk of falls and accidental injuries from falls in elderly people with sarcopenia.

Fall prevention is not only related to acute proprioception and adequate muscle strength but also depends on the neuromuscular response, that is, the timely activation of the appropriate postural response to control the body’s center of gravity once displacement occurs (14). Neuromuscular response is also considered to be one of the most important factors contributing to falls (15). Older adults exhibit greater deficits in neuromuscular responses, leading to a potentially slower correction of postural disturbances, ultimately increasing their incidence of falls, which is more pronounced in elderly patients with sarcopenia (16). In particular, for the aged population, exercise intervention is a powerful approach to enhance neuromuscular function (15). However, there is no clear consensus on appropriate exercise interventions for sarcopenia. The gentle, beautiful motions of Tai Chi, a traditional Chinese workout regimen, are what make it so popular. In China, Tai Chi practice is widespread and has higher compliance rates in older persons than simple resistance exercise (17). Studies have revealed that those who practice Tai Chi have greater muscle strength, balance, coordination, and concentration abilities than control groups (18–21). These qualities help to improve physical function and avoid falls in older adults. The processes by which Tai Chi elicits these improvements are yet unknown, although Tai Chi is recognized as a useful method for improving balance and reducing falls. One study showed that older adults who regularly participated in Tai Chi had more sensitive neuromuscular responses than sedentary controls and responded more quickly to surprise ankle inversion perturbations, which facilitated the timely correction of postural problems to avoid falls (22). However, no studies have elucidated the potential mechanisms by which Tai Chi improves the dynamic stability of elderly patients with sarcopenia, especially the association with neuromuscular responses. Therefore, this study examined the effects of 12 weeks of regular Tai Chi practice on neuromuscular responses in elderly patients with sarcopenia and further investigated the mechanisms by which Tai Chi practice improves their postural control. In this study, we hypothesize that 12 weeks of Tai Chi exercise can improve the neuromuscular reactions and dynamic postural control ability of elderly patients with sarcopenia and that there is a certain correlation between the degrees of improvement in the two.

## Methods

This study was designed as a parallel randomized controlled trial. The study protocol was conducted in accordance with the Declaration of Helsinki and was approved by the Medical Ethics Committee of Shanghai Fourth People’s Hospital (No. SYLL2023008). We registered the study in the Chinese Clinical Trial Registry (No. ChiCTR2200063921).

### Participants

Participants were recruited in the Outpatient Department of ZheJiang Hospital and surrounding communities through posters, oral presentations, Internet advertisements, and WeChat platforms from March 2021 to September 2022. From the study of Xu et al. (22), the reaction time of the tibialis anterior muscle was 81.91 ± 8.20 ms in older adults with Taijiquan intervention and 88.52 ± 6.67 ms in those without specific exercise intervention. The sample size was calculated by analysis using PASS software (power = 0.86; *α* = 0.05). The minimum sample size needed was 25, and considering a 15% shedding rate, the sample size required for each group in this study was 30. Inclusion criteria were as follows: (1) met the diagnostic criteria for sarcopenia (Asian Working Group for Sarcopenia, AWGS) (2); (2) male or female participants aged 60 to 80 years; (3) had a history of falling during the last 2 years and met the cutoff time of 15.96 s for the Timed Up & Go (TUG) test to check for recurrent falls; (4) were able to walk independently without the help of an aid, such as a cane; and (5) agreed to a random assignment to receive an intervention for 12 weeks and to sign an informed consent form. Exclusion criteria were as follows: (1) sprains, fractures, severe joint deformities or other traumatic injuries that cause immobility; (2) severe cardiovascular or cerebrovascular diseases, skeletal muscular system diseases or neurological diseases; (3) speech, hearing, cognitive or vestibular dysfunction; (4) history of regular exercise within the last 1 year; and (5) taking part in additional forms of exercise during the research period.

**Figure 1:**
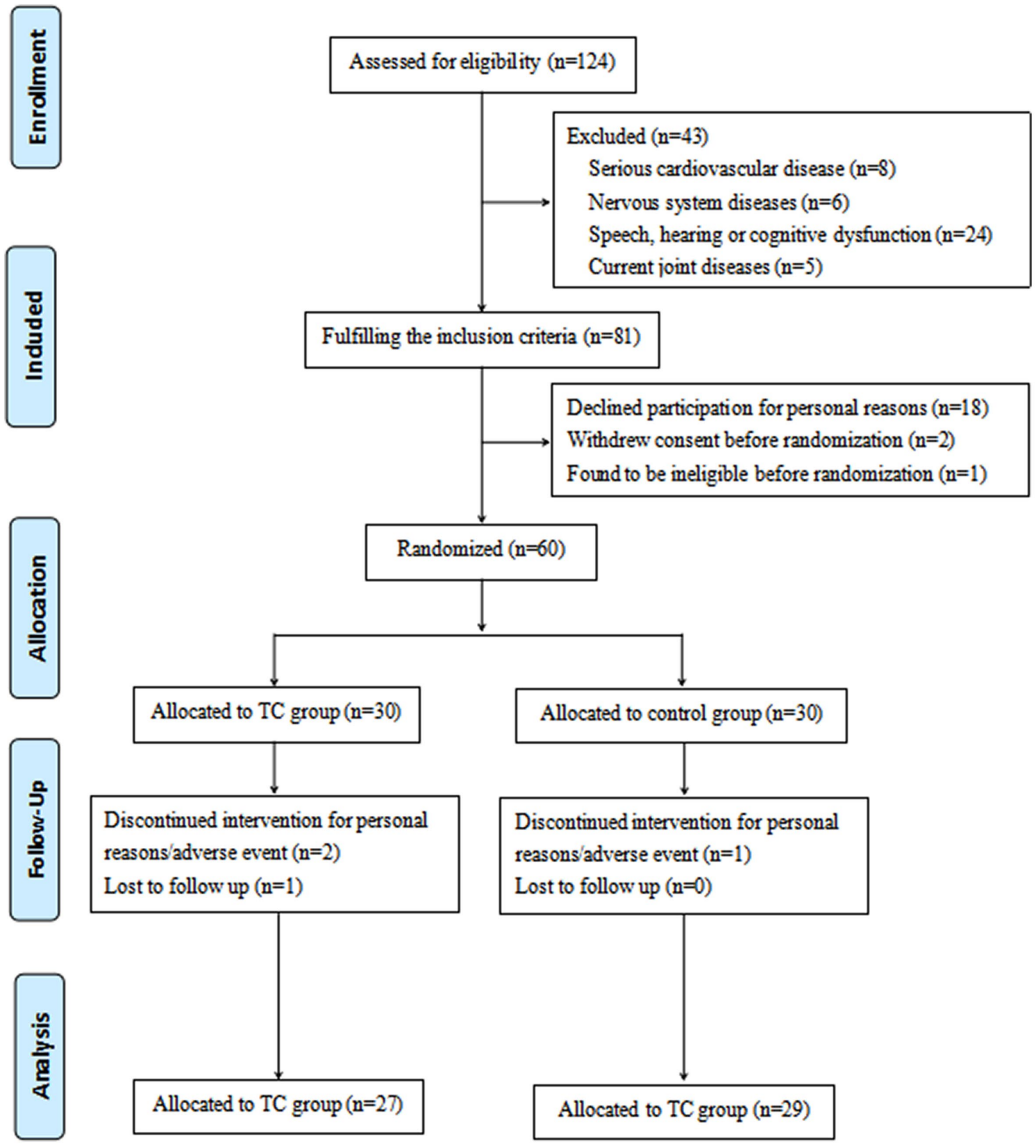
CONSORT diagram of the eligibility, exclusion and randomization scheme.

Initially, 124 participants were selected, however, 64 were later disqualified. In particular, 43 participants did not meet the inclusion requirements, and 21 people were unable to finish the entire study for a variety of reasons. The remaining 60 participants met the qualifying requirements and completed the informed consent form (Figure 1).

### Randomization and blinding

A total of 60 eligible participants were randomly assigned to either the Tai Chi group (*n* = 30) or the control group (*n* = 30). An independent researcher who was not involved in baseline data collection, clinical interventions, outcome evaluations, data gathering, or statistical analysis used the SAS Statistics program version 9.4 (SAS Institute Inc. Cary, NC, United States) to generate random sequences. Assignments were enclosed in sequentially numbered, opaque-sealed envelopes to hide the participant allocation. Clinicians strictly followed the aforementioned inclusion and exclusion criteria when deciding whether subjects were eligible to participate in the study. The eligible number of patients was sent to the recruiters by the researchers, who also obtained a sealed, opaque packet with information about the intervention, allocation, and randomization. Then, they gave the envelope to the therapist or clinician. Notably, group allocations were not disclosed to the researchers in charge of recruiting subjects. The clinical intervener opened the envelope and learned about the intervention plan that was given to the participants. The participants, clinician, and therapist could not be blinded after being assigned to the clinical intervention due to its nature. Prior to the completion of all data analyses, the outcome assessors and data statisticians did not know the treatment allocation.

### Interventions

#### The control group

Starting at the beginning of the study, participants in the control group received 45-min health education sessions once every 2 weeks for 12 weeks. Health education was conducted by a clinician to educate the participants regarding sarcopenia, including the etiology, pathogenesis, clinical manifestations, hazards to elderly individuals, and prevention and treatment measures of sarcopenia. Participants in the control group maintained their prestudy routine during the experimental period and did not participate in any planned training activities, such as brisk walking or resistance training.

#### The Tai Chi group

The Tai Chi group engaged in a 40-min simplified eight-style Tai Chi exercise session based on the provided health education. The simplified eight-style Tai Chi exercise procedures included (1) brachial rewinding, (2) left- and right-knee kyphosis steps, (3) left and right mustang mane, (4) cloud hand, (5) left and right Golden Rooster, (6) right and left foot pedal, (7) right- and left-wing fingertips, and (8) crossing hands. Simplified eight-style Tai Chi is composed of slow, fluid, and rhythmic motions that highlight the practitioner’s attention on trunk rotation, weight shifting, coordination, and maintenance of lower limb postural stability. To prevent overly rigorous activities around the knee joints, changes were made that concentrated on decreasing and avoiding sustained unilateral weight bearing, dynamic rotation of the knee joints, and excessive knee flexion (i.e., low stance). The training program started with simple standing postures that emphasized proper body alignment, body mass centering, weight shifting in multiple directions, and easy knee flexion and extension with minimal resistance. The difficulty level of the exercises gradually increased over time.

The protocol comprised three distinct phases, wherein the first phase (weeks 1–2) emphasized basic Tai Chi preparatory movement exercises such as weight shifting, knee flexion, push-off with toes, meditation, rhythmic breathing, and Tai Chi, with 4 repetitions of 5 min with 3 min of recovery between repetitions. The second phase (weeks 3–4) centered on mastering the forms and associated movements, with 5/6 repetitions of 4 min with 2 min of recovery between repetitions. The third phase (weeks 5–12) focused on practicing and reinforcing the precision and sequence of the forms by varying the practice configuration, such as changing directions, 7/8 repetitions of 3 min with 1 min of recovery between repetitions.

An experienced Tai Chi coach instructed and supervised patients with sarcopenia to maintain the consistency of the practice time and the fundamental correctness of the action rhythm. Each tai chi session included three components: a 5-min warm-up, a 30-min tai chi exercise routine, and a 5-min cool-down. There were 36 sessions held three times per week over the course of 12 weeks.

Patients were encouraged to participate even if they experienced slight fatigue throughout the entire training process. If a patient’s fatigue was obvious during a training session, as measured by a Borg’s CR-10 scale score of >4 points, we advised the patient to rest or stop training and resume once the fatigue had been relieved.

#### Outcome measurements

Two assessors who had received professional training and were unaware of the intervention allocation assessed the subjects within 3 days prior to the intervention and within 3 days after completion of the intervention.

Referring to the designs reported in other related studies (23, 24), we simulated ankle inversion using the movable platform offered by the dynamic stability test module in ProKin 254 (TecnoBody, Italy). The foot platform on this device can tilt up to 15° from horizontal on all sides. The tilting’s onset and terminating signals could be collected simultaneously with the EMG signals.

First, the tests were explained to the participants, after which the equipment was calibrated for each person’s age, height, and body mass. The test subjects were instructed to stand on the platform in bare feet, evenly distributing their weight between the two feet and arms crossed against chest. The axis of rotation of the platform was just medial to between the two feet. To familiarize the subjects to the dynamic stability testing procedure, they were given two practice runs on the movable platform.

Then, the placement of the EMG electrodes above the center of the rectus femoris, semitendinosus, anterior tibialis, and gastrocnemius of the right leg was confirmed by manual testing and voluntary contractions. After the subject stood on the platform, the researcher turned on the dynamic stability test without the subject’s knowledge to make the platform movable, and the researcher simultaneously randomly pressed a plane on the platform to cause a sudden perturbation of the subject’s ankle joint. Subjects were required to regain balance through lower limb control and remain on the movable platform for 20 s. At the end of the test, we obtained the overall stability index (OSI) of each subject by ProKin 254, and the EMG activity of the right leg muscles was collected.

Subjects who shifted their feet on the platform or held the bar with both hands during the test were considered to have failed the test and needed to retest. At least three qualified EMG data sets were collected for each subject. Rest periods of approximately 2 min in length were provided between trials to counteract the risk of subject fatigue.

#### Overall stability index

The OSI reflects the subject’s postural control, with larger values indicating poorer postural control and higher fall risk (25).

#### EMG (neuromuscular response time)

The surface EMG signals of the rectus femoris, semitendinosus, anterior tibialis, and gastrocnemius muscles of the right leg of each individual were collected using a Bagnoli-8 EMG system (Delsys, United States). LabVIEW Software (National Instruments, United States) was used to sample the raw EMG signals at 1000 Hz, and the results were saved on a computer for off-line data reduction.

A similar procedure to that used by Xu et al. (22) and Li et al. (26) was used for EMG data reduction. EMG software was used to measure the raw EMG signals. The period in milliseconds (1 sample = 1 ms) between the initiation of the movable platform and the first rising response of EMG signals from baseline to certain activity is referred to as the neuromuscular response time. A tiny artifact occasionally appeared in the EMG signal when the electric motor operating the movable platform started. The earliest reflex activity would start 45 ms after the movable platform opened; therefore, everything that happened before that was disregarded. The same researcher analyzed the data from the right leg.

### Statistical analysis

Statistical analysis was carried out using SPSS Statistics, version 22.0 (SPSS Inc., Chicago, IL, United States). The intention-to-treat principle was used for all assigned individuals with data to analyze the results. If the distribution of the variables was normal or skewed, continuous variables were expressed as the mean standard deviation (SD) or medians (25th to 75th centiles). The Mann–Whitney test was used for further analysis of categorical data. Repeated-measures ANOVA was used to evaluate variables with multiple measurements. To clarify the relationship between neuromuscular response and postural control ability, we calculated Pearson’s or Spearman’s correlation coefficients (*r*) between 12-week changes in the response time of the rectus femoris, semitendinosus, anterior tibialis, and gastrocnemius muscles of the right leg and OSI. Statistical significance was determined when a result had a corresponding *p-*value <0.05.

## Results

At 12 weeks, 56 of the 60 eligible individuals had finished the assessment. After several weeks of exercise, a total of 3 participants in the Tai Chi group and 1 participant in the control group dropped out, and contact was lost. After 12 weeks, participants in the Tai Chi group and control group completed 90 and 96.7%, respectively, of the total planned exercise sessions (Figure 1). As far as the baseline comparison allowed, there were no differences between the two participant groups (Table 1).

**Table 1:** Baseline demographic and clinical characteristic of the participants.

Demographic characteristic	Tai Chi group (*n* = 27)	Control group (*n* = 29)	*t*/*χ*^2^	*p*
Age (year)	69.70 ± 5.05	72.14 ± 4.79	−1.852	0.07
Gender			1.220	0.269
Male	17	14		
Female	10	15		
Height (cm)	163.11 ± 6.79	162.24 ± 5.14	0.540	0.591
Weight (kg)	55.08 ± 2.57	54.53 ± 1.85	−0.599	0.549
BMI (kg/m^2^)	20.75 ± 1.23	20.74 ± 0.80	0.037	0.970
Education			1.259	0.739
Bachelor or above	3	5		
High school	7	8		
Middle school	5	7		
Primary school	12	9		
Number of fall			0.386	0.534
>2 times per year	9	12		
<2 times per year	18	17		
Fall injury	8	11	0.430	0.512
TUGT (second)	20.63 ± 3.31	19.11 ± 2.52	−1.600	0.110

Within-group comparisons revealed that in the Tai Chi group, there was a significant reduction in OSI and response time of the rectus femoris, semitendinosus, anterior tibialis, and gastrocnemius after 12 weeks of intervention compared to baseline (*p* < 0.05), but there were no significant prepost differences in the above indicators in the control group (*p* > 0.05). Between-group comparisons demonstrated no significant baseline differences between the Tai Chi and control groups for OSI or response time of the rectus femoris, semitendinosus, anterior tibialis, and gastrocnemius (*p* > 0.05), but the Tai Chi group had significantly lower levels of these indicators than the control group after treatment (*p* < 0.05) (Tables 2, 3).

**Table 2:** Comparison of reaction time at baseline and 12-week follow-up among the two groups (mean ± standard deviations).

Outcome variable	Tai Chi group (*n* = 27)	Control group (*n* = 29)
Rectus femoris		
Baseline	142.75 ± 8.41	141.03 ± 7.00
After 12-week intervention	143.47 ± 7.17	126.25 ± 7.58^ab^
Semitendinosus		
Baseline	138.07 ± 5.56	137.36 ± 7.19
After 12-week intervention	137.14 ± 4.17	127.10 ± 7.54^ab^
Gastrocnemius		
Baseline	151.04 ± 6.55	150.28 ± 5.87
After 12-week intervention	152.30 ± 5.73	137.36 ± 6.26^ab^
Anterior tibialis		
Baseline	135.40 ± 6.34	134.17 ± 5.52
After 12-week intervention	136.64 ± 6.28	121.28 ± 5.81^ab^

^a^Denotes a difference significant at *p* < 0.05 when compared with pretest values; ^b^denotes a difference significant at *p* < 0.05 when compared with the control group.

**Table 3:** Comparison of OSI at baseline and 12-week follow-up among the two groups (mean ± standard deviations).

Outcome variable	Tai Chi group (*n* = 27)	Control group (*n* = 29)
Baseline	4.65 ± 0.42	4.80 ± 0.29
12-week	4.76 ± 0.35	4.00 ± 0.24^ab^

^a^Denotes a difference significant at *p* < 0.05 when compared with pretest values; ^b^denotes a difference significant at *p* < 0.05 when compared with the control group.

In the Tai Chi group, significantly positive correlations were observed between the changes in OSI and the changes in response time of the rectus femoris (*r* = 0.682, *p* < 0.001), semitendinosus (*r* = 0.488, *p* = 0.007), anterior tibialis (*r* = 0.757, *p* < 0.001), and gastrocnemius (*r* = 0.767, *p* < 0.001). Furthermore, no significant correlations were observed between changes in OSI and response times in the control group (*p* > 0.05) (Table 4).

**Table 4:** Correlations of changes in reaction time with changes in OSI among Tai Chi Group (*n* = 27) and control group (*n* = 29).

	OSI	
	*r*	*p*
Reaction time (rectus femoris)		
Control group	−0.142	0.480
Tai Chi group	0.682	0.000
Reaction time (semitendinosus)		
Control group	0.121	0.547
Tai Chi group	0.488	0.007
Reaction time (anterior tibialis)		
Control group	−0.015	0.940
Tai Chi group	0.757	0.000
Reaction time (gastrocnemius)		
Control group	0.193	0.334
Tai Chi group	0.767	0.000

## Discussion

Our 12-week Tai Chi training protocol resulted in a significant reduction in neuromuscular response time for the right lower limb muscles of the rectus femoris, semitendinosus, gastrocnemius, and anterior tibialis in elderly patients with sarcopenia. Additionally, the patients’ dynamic posture control ability was also significantly improved. Sorock et al. believe that the deterioration of neuromuscular function related to age or disease is the root cause of increased risk of falls in the elderly (27). There is currently research indicating that elderly individuals have longer neuromuscular response times in their lower limbs than younger individuals (28–30). Regarding the differences between fallers and nonfallers, Studenski et al. found that fallers have a 7–10 ms longer neuromuscular response time as well and believe that excessively delayed neuromuscular responses can lead to inadequate postural adjustments in situations of falling (31). Therefore, observing a shortened neuromuscular response time and improved performance on dynamic balance tests in our study indicates that our 12-week Tai Chi training protocol has a positive effect on improving postural control and reducing the risk of falls in elderly patients with sarcopenia. Furthermore, our study found a positive correlation between the degree of improvement in neuromuscular response and dynamic posture control ability, which further reflects the potential role of enhanced neuromuscular response as a factor in improving posture control and reducing the risk of falls in elderly patients with sarcopenia through Tai Chi exercise.

Tai Chi is a graceful, slow, continuous movement form that demands that exercisers always maintain flexion of the hip and knee and dorsiflexion of the ankle during exercise. Sun et al. found that after 1 year of Tai Chi exercise in elderly women, the neuromuscular response times of the rectus femoris, semitendinosus, and gastrocnemius muscles were significantly shortened (32). Similarly, DQ also demonstrated that long-term Tai Chi exercise has a positive effect on the neuromuscular response of the lower limbs (22). However, there are currently no studies reporting the effect of Tai Chi on neuromuscular response in elderly patients with sarcopenia. Possible reasons include the following: (1) each movement of Tai Chi requires accuracy, fluidity, and naturalness, but Tai Chi involves much single-leg support and significant weight shifting movements, which can be difficult for elderly patients with sarcopenia to complete; and (2) traditional Tai Chi training typically consists of 24 movements, which can be time-consuming and may pose a challenge for elderly patients with sarcopenia to complete the entire sequence. Taking into account the above reasons, we have adopted the simplified eight-style Tai Chi exercise, which reduces and avoids continuous single-leg weight-bearing, dynamic rotation of the knee joint, and excessive flexion of the knee joint, and have developed a progressive training plan. From the results of our study, the rectus femoris, semitendinosus, anterior tibialis, and gastrocnemius muscles of the patients in the Tai Chi group showed faster responses than those of the patients in the control group after 12 weeks of intervention, and further comparison of dynamic posture control ability revealed that the OSI in the Tai Chi group was better than that in the control group after 12 weeks of intervention. This is related to the ability of Tai Chi to improve neuromuscular responses in elderly patients with sarcopenia. First, long-term Tai Chi exercise can frequently stimulate joint proprioceptors, promote and consolidate the process of proprioceptive transmission, and strengthen the neuromuscular response ability, and when the body is disturbed by the outside world, it can quickly perceive changes in body space position and maintain balance. Second, in Tai Chi, participants always maintain a squatting position with flexed hips and bent knees with their own body weight, providing the resistance to keep the lower limb muscles in a state similar to a constant isometric muscle action. This improves the central nervous system excitation time, which is conducive to neuromuscular response ability.

When an organism is subjected to external environmental disturbances, the central nervous system takes the lead in producing preprogrammed posture responses, including feedforward control, feedback control, and voluntary movement control, which sequentially activate posture muscle activity, action muscle activity, and the coordination between the two, thereby quickly and effectively maintaining body center of gravity stability and limb spatial positioning and preventing imbalance and falls. To some extent, the neuromuscular response ability in elderly people can represent their posture control ability. After sudden ankle perturbation, the loaded leg displayed flexion of the hip and knee and dorsiflexion of the ankle (26). Obviously, this is accomplished by contracting the anterior tibialis, gastrocnemius, hamstrings and quadriceps muscles. This activation strategy appears to have two goals: to increase stability and to minimize the load on the inverting foot. Therefore, in this study, we reflected the patient’s posture control capability by analyzing the neuromuscular response ability of the aforementioned four muscles.

A previous study on upright subject posture reactions typically employed the classic trap-door test using ankle inversion devices with a 30° tilted trapdoor to induce ankle inversion, combined with surface electromyography measures of muscle activation timing, activation duration, and muscle activation level (23, 33, 34). However, for elderly patients with sarcopenia, the 30° tilted angle is often too large, and they may have difficulty completing the test or even fall during the test. Moreover, this ankle inversion device cannot objectively reflect the patient’s overall postural control during testing. Finally, in daily life, patient falls are caused not only by ankle inversion but also by ankle eversion, ankle dorsiflexion, and ankle plantarflexion in other directions. In our study, we chose the unstable platform provided by the dynamic stability test module in ProKin 254 to observe the pose response. ProKin254, as a new type of balance testing device, utilizes computerized numerical assessment to provide accurate quantitative scores, which can comprehensively, in detail, and objectively evaluate the posture control ability of the subjects. The dynamic stability test module in Prokin254 enables the platform to tilt in various directions to simulate scenarios of patients falling in different directions. Meanwhile, considering the particulars of elderly patients with sarcopenia, the tilting angle was decreased to 15° to prevent falls during the test. In this study, we used Prokin254 to assess the postural control ability of subjects under sudden ankle disturbance and real-time evaluation of the neuromuscular response ability of the rectus femoris, semitendinosus, anterior tibialis, and gastrocnemius and explored the potential correlation between the two. The results showed that the degree of improvement in neuromuscular response time in the Tai Chi group was significantly positively correlated with the degree of improvement in postural control ability, suggesting that Tai Chi may improve postural control ability and reduce the risk of falls in elderly patients with sarcopenia by improving neuromuscular response ability.

## Conclusion

Our Tai Chi training protocol simplifies the training movements, reduces the training difficulty, and gradually increases the workload. This training protocol is feasible, safe, and repeatable for elderly patients with sarcopenia. Twelve-weeks of Tai Chi exercise can improve the neuromuscular response of the lower extremities in elderly patients with sarcopenia, shorten their neuromuscular response time when balance is endangered, and enhance their dynamic posture control ability. The application of this protocol in communities may favor a reduction in fall risk and promote socialization among elderly patients with sarcopenia. In the future, we plan to conduct multicenter, large-sample size studies to further validate the conclusion of this study, thus promoting the promotion and application of this protocol among elderly patients with sarcopenia.

## Data availability statement

The raw data supporting the conclusions of this article will be made available by the authors, without undue reservation.

## Ethics statement

The studies involving human participants were reviewed and approved by the Medical Ethics Committee of Zhejiang Hospital (No. 2021-135 K). The patients/participants provided their written informed consent to participate in this study.

## Author contributions

DH designed the clinical trial, analyzed the data, and wrote the publication. XK, WS, JF, and AZ carried out the experiment’s feasibility analysis, were in charge of the article’s quality control and review, and were in charge of the article’s general management and supervision. HZ, RZ, and FL were responsible for data gathering and trial evaluation. CJ provided support for language polishing and guidance for final revisions of the article. All authors reviewed and approved the article’s submission.

## Funding

This work was supported by the Research Launch Project of the Fourth People’s Hospital Affiliated to Tongji University (Grant Number: sykyqd02001) and the Zhejiang Province excellent Young talents Fund Project of traditional Chinese Medicine (Grant Number: 2022ZQ004).

## Conflict of interest

The authors declare that the research was conducted in the absence of any commercial or financial relationships that could be construed as a potential conflict of interest.

## Publisher’s note

All claims expressed in this article are solely those of the authors and do not necessarily represent those of their affiliated organizations, or those of the publisher, the editors and the reviewers. Any product that may be evaluated in this article, or claim that may be made by its manufacturer, is not guaranteed or endorsed by the publisher.
